# Transcriptional Regulation: Molecules, Involved Mechanisms, and Misregulation

**DOI:** 10.3390/ijms20061281

**Published:** 2019-03-14

**Authors:** Amelia Casamassimi, Alfredo Ciccodicola

**Affiliations:** 1 Department of Precision Medicine, University of Campania “Luigi Vanvitelli”, Via L. De Crecchio, 80138 Naples, Italy; 2 Institute of Genetics and Biophysics “Adriano Buzzati Traverso”, CNR, 80131 Naples, Italy; 3 Department of Science and Technology, University of Naples “Parthenope”, 80143 Naples, Italy

Transcriptional regulation is a critical biological process that allows the cell or an organism to respond to a variety of intra- and extra-cellular signals, to define cell identity during development, to maintain it throughout its lifetime, and to coordinate cellular activity. This highly dynamic mechanism includes a series of biophysical events orchestrated by a huge number of molecules establishing larger networks and occurring through multiple temporal and functional steps that range from specific DNA-protein interactions to the recruitment and assembly of nucleoprotein complexes. Essentially, the key transcription levels include the recruitment and assembly of the entire transcription machinery, the initiation step, pause release and elongation phases, as well as termination of transcription. Additionally, these steps are interconnected with governing chromatin accessibility (such as the unwrapping process, which is controlled by histone modification and chromatin remodeling proteins), and other epigenetic mechanisms (such as enhancer-promoter looping, which is necessary for a successful gene transcription). Finally, various RNA maturation events, such as the splicing that occurs with transcription, constitute an additional level of complexity. Numerous molecules and molecular factors, including transcription factors, cofactors (both coactivators and corepressors), and chromatin regulators, are known to participate to this process [1]. Essential components of the basal transcription machinery comprise the RNA polymerase II holoenzyme, the general initiation transcription factors (TFIIA, -IIB, -IID, -IIE, -IIF, and -IIH) and the Mediator complex, a multi-subunit compound that joins transcription factors bound at the upstream regulatory elements—such as nuclear receptors—and all the remaining apparatus at the promoter region. It is noteworthy that it also works in close interplay between the basal machinery and factors responsible for the epigenetic modifications; for instance, together with cohesin, it facilitates DNA looping [2]. More recently, a novel multi-subunit complex named Integrator was added as one of the components of the RNA Polymerase II-mediated transcription apparatus. It is also involved in many stages of eukaryotic transcription for most regulated genes [3].

Additionally, the high complexity of transcriptional regulation is also derived from the involvement of non-coding RNAs (ncRNAs). Indeed, research over the last two decades has revealed new classes of ncRNAs, including microRNAs (miRNAs), small nucleolar RNAs (snoRNAs), long ncRNAs (lncRNAs), circular RNAs (circRNAs), and enhancer RNAs (eRNAs), each with different regulatory functions and altogether belonging to a larger RNA communication network ultimately controlling the production of the final protein [4].

Recent advances in “omics” and computational biology have provided novel tools that allow one to integrate different layers of information from biophysical, biochemical, and molecular cell biology studies. In turn, these novel strategies provided a fuller understanding of how DNA sequence information, epigenetic modifications, and transcription machinery cooperate to regulate gene expression. Of note, most of the new molecular biomarkers and therapeutic targets for several human pathologies derive from transcriptome profiling studies, and their number is continuously increasing. Next Generation Sequencing (NGS), mainly RNA-Sequencing (RNA-Seq), has completely revolutionized transcriptome analysis, allowing the quantification of gene expression levels and allele-specific expression in a single experiment, as well as the identification of novel genes, splice isoforms, fusion transcripts, and the entire world of ncRNAs at an unprecedented level [4].

It is well known that many human disorders are characterized by global transcriptional dysregulation because most of the signaling pathways ultimately target transcription machinery. Indeed, many syndromes and genetic and complex diseases—cancer, autoimmunity, neurological and developmental disorders, metabolic and cardiovascular diseases—can be caused by mutations/alterations in regulatory sequences, transcription factors, cofactors, chromatin regulators, ncRNAs, and other components of transcription apparatus [1,2,3,4]. Thus, advances in our understanding of molecules and mechanisms involved in the transcriptional circuitry and apparatus lead to new insights into the pathogenetic mechanisms of various human diseases and disorders.

In this special issue, a total of 19 excellent and interesting papers consisting of 11 original research studies, seven reviews, and one communication are published [5,6,7,8,9,10,11,12,13,14,15,16,17,18,19,20,21,22,23]. They cover all subjects of transcriptional regulation, from cis-regulatory elements to transcription factors, chromatin regulators, and ncRNAs. Additionally, several transcriptome studies and computational analyses are also included in this issue.

Huang et al. analyzed the transcriptional regulation of the gene coding for the Chloride intracellular channel 4 (*CLIC4*). This is a multifunctional protein with diverse physiological functions. Differential expression of *CLIC4* between cancer cells and the surrounding stroma has been reported in various tumor types [11]. Here, the authors found an alternative G-quadruplex (G4) structure, PG4-3, in its promoter region. Through the use of the CRISPR/Cas9 system, they provided evidence that this element could play an important role in regulating the *CLIC4* transcription levels [11].

Regarding transcription factors, a comprehensive review summarized the structures and functions of these regulators in both model and non-model insects, including Drosophila, and appraises the importance of transcription factors in orchestrating diverse insect physiological and biochemical processes [17]. An original article examined the paired-box 3 (*Pax3*) transcription factor in the winged pearl oyster *Pteria penguin*. More precisely, this study investigated the role of *PpPax3* in melanin synthesis and used RNA interference to provide evidence that this function is exerted in this important marine species through the tyrosinase pathway [18]. A bioinformatics approach was used to identify the significant genes responsible for the human Patau syndrome (PS), a rare congenital anomaly due to chromosome 13 trisomy. This molecular network analysis and protein-protein interaction study indicated *FOXO1* (Forkhead Box O1) as a strong transcription factor interacting with other key genes associated with lethal heart disorders in PS. [15].

As expected in the NGS era, transcriptome analysis by RNA-Seq has been widely used in many studies to elucidate the most varied mechanisms of pathophysiology as well as other relevant biological processes in diverse organisms [5,9,20,21]. Actually, a small number of studies still utilize microarray as a useful approach. Indeed, this platform allows one to identify the common pathway(s) of Major Depressive Disorder and glioblastoma [5]. Otherwise, most of the studies employ RNA-Seq to, for example, understand the regulatory system of stringent response in sphingomonads [9] or to unravel molecular insights of phase-specific pollen-pistil interaction during self-incompatibility and fertilization in tea [21]. Additionally, in silico analyses of available transcriptome databases are often very useful when the biological material is scarce or difficult to isolate, as in the case of a study aimed to identify genes that could have a potential role in the oyster larval adhesion at the pediveliger stage [20]. Additionally, the availability of multi-omics datasets from patient tissues represents a unique source to study human diseases. Particularly, The Cancer Genome Atlas (TCGA) collects data from thousands of subjects with human malignancies, thus enabling the in silico analysis of genes or families of genes of interest. For example, in an effort to obtain a pan-cancer overview of the genomic and transcriptomic alterations of the PR/SET domain gene family (PRDM) members in cancer, our group reanalyzed the Exome- and RNA-Seq datasets from the TCGA portal [12]. Likewise, to date, a lot of similar studies have led to a better comprehension of the pathogenetic mechanisms as well as the discovery of novel biomarkers and/or therapeutic targets for these human disorders, as cited in a review dissecting the role of Adiponectin as a link factor between adipose tissue and cancer [23].

In the field of cancer research, an interesting pathogenetic mechanism involving dysregulation of transcription is represented by the destabilization of the messenger RNAs of critical genes implicated in both tumor onset and tumor progression exerted by tristetraprolin (TTP). Indeed, as reviewed in a paper of this special issue, the tumor suppressor TTP can negatively regulate tumorigenesis. In turn, TTP expression is frequently downregulated in several tumors by various mechanisms [13].

Several papers have described novelties in the field of ncRNAs. For instance, a study investigated the possible role in cell metabolism of miR-25-3p. This miRNA is highly conserved in mammals and was previously found to be involved in many biological processes and in some cancer and cardiovascular related diseases. Specifically, in the C2C12 cell line derived from mouse muscle myoblasts, it is positively regulated by the transcription factor AP-2α and enhances cell metabolism by directly targeting the 3′ untranslated region of AKT serine/threonine kinase 1 (*Akt1*), a gene related to metabolism [6].

LncRNAs play an important role as epigenetic and transcriptional regulators. Evidence of their importance in the pathophysiology of many malignancies has drastically increased in the last decade. In their excellent contribution, Cruz-Miranda et al. describe the functional classification, biogenesis, and role of lncRNAs in leukemogenesis, highlighting the evidence that lncRNAs could be useful as biomarkers in the diagnosis, prognosis, and therapeutic response of leukemia patients, as well as showing that they could represent potential therapeutic targets in these tumors [22]. In a preliminary study, RNA-Seq data were used to profile, quantify, and classify (for the first time) lncRNAs in human term placenta [8]. Although the obtained lncRNAs still need to be functionally characterized, they could expand the current knowledge of the essential mechanisms in pregnancy maintenance and fetal development.

Lei et al. proposed a new computational path weighted method for predicting circRNA-disease associations, the PWCDA method. Despite some limitations, it showed a much better performance than other computational models [14].

A remarkable study explored the utility of eRNA expression as a causal anchor in predicting transcription regulatory networks based on the observation that eRNAs mark the activity of regulatory regions [16]. In their work, the authors developed a novel statistical framework to infer causal gene networks (named Findr-A) by extending the Findr software for causal inference through the use of cap analysis of gene expression (CAGE) data from the FANTOM5 consortium [16].

Numerous epigenetic mechanisms other than regulation by ncRNAs take place during RNA polymerase II-transcription and may be involved in human pathophysiology. An outstanding review on the Cyclin Dependent Kinase Inhibitor 1C (*CDKN1C*) gene summarizes all the possible (epi)-genetic alterations leading to diseases. This gene encodes the p57Kip2 protein, the third member of the CIP/Kip family, and its alterations are known to cause three human hereditary syndromes characterized by altered growth rate. Interestingly, *CDKN1C* is positioned in a genomic region characterized by a remarkable regional imprinting that results in the transcription of only the maternal allele. Moreover, this gene is also down-regulated in human cancers. Of note, its transcriptional regulation is linked to several mechanisms, including DNA methylation and specific histone modifications. Finally, ncRNAs also play important roles in controlling p57Kip2 levels [7].

Selenium-related transcriptional regulation is the topic of a comprehensive review [10]. Selenium is a trace element controlling the expression levels of numerous genes; it is essential to human health, and its deficiency is related to several diseases. It is incorporated as seleno-cysteine to the so-called seleno-proteins via an uncommon mechanism. Indeed, the codon for seleno-cysteine is a regular in-frame stop codon, which can be passed by a specific complex translation machinery in the presence of a signal sequence in the 3′-untranslated part of the seleno-protein mRNAs. Nonsense-mediated decay and other mechanisms are able to regulate seleno-protein mRNA levels [10].

It is well-known that DNA methylation contributes to the gene expression regulation without changing the DNA sequence. Abnormal DNA methylation has been associated with improper gene expression and may lead to several disorders. Both genetic factors and modifiable factors, including nutrition, are able to alter methylation pathways. An interesting review of this special issue carefully describes molecular mechanisms underlying the link between diet and DNA methylation [19].

Finally, we hope the readers enjoy this Special Issue of IJMS and the effort to present the current advances and promising results in the field of transcriptional regulation and its involvement in all of the relevant biological processes and in pathophysiology.
